# Live-cell imaging and analysis of 3D spheroids in hypoxia- and radiotherapy-related research

**DOI:** 10.1016/j.ctro.2025.100920

**Published:** 2025-01-15

**Authors:** Claire Beckers, Lazaros Vasilikos, Lorena Moor, Martin Pruschy

**Affiliations:** Laboratory for Applied Radiobiology, Department of Radiation Oncology, University Hospital Zurich, University of Zurich, Zurich, Switzerland

**Keywords:** Spheroids, Radiotherapy, Hypoxia, Fluorescence microscopy, Flow cytometry, 3D cell culture

## Abstract

3D spheroids are a valuable tool for investigating the interplay between hypoxia and radioresistance. However, standardized methods for visualizing normoxic and hypoxic regions within spheroids and assessing treatment responses are lacking. We developed a straightforward workflow for the analysis of spheroids based on image processing using a novel custom-written script, and flow cytometry.

## Highlights

• 3D spheroids are a valuable tool for hypoxia and radiotherapy research.
• Live-cell imaging of normoxic, hypoxic, and necrotic regions offer treatment response insights.
• Image analysis and flow cytometry link quantitative data with spatially differential treatment responses.

## Introduction

3D spheroids are a physiologically more relevant in vitro model than tumor cells grown in 2D monolayers. Spheroids are representative for solid tumors in the sense that they consist of a proliferative outer rim, followed by a quiescent and hypoxic zone and ultimately a necrotic core as cells are progressively distanced from adequate nutrient and oxygen supply [1], [2], [3]. Furthermore, they are cost-effective, more time efficient and bypass ethical concerns in comparison to in vivo experiments [4], [5]. In radiotherapy research, 3D spheroids can also serve as a valuable tool to study hypoxia-induced radioresistance [6], [7], [8]. Despite extensive research dedicated to identifying treatment modalities that target hypoxic tumor cells as part of combined treatment with radiotherapy, the translation of such approaches into clinical settings have remained limited [9], [10]. There is thus an urgent need for more efficient and standardized tools to explore treatment options that can overcome hypoxia-induced radioresistance at the preclinical level to ensure a more successful translation into the clinics [3], [11], [12]. The challenge with employing 2D cell culture in radiotherapy and hypoxia research lies in the requirement for complex installations such as hypoxic chambers and irradiation equipment that enable treatment under sustained hypoxic conditions without causing reoxygenation during transport to the irradiation facility. These challenges can be overcome by using 3D spheroids that inherently have a hypoxic core.

However, visualization of the hypoxic core and quantification of a treatment response in spatially distinct regions within a 3D spheroid represent an experimental challenge. Here, we address this challenge by providing a simple and standardized method for spheroid seeding and live-cell imaging that can be carried out with basic cell culture equipment, and a novel custom-written script for the analysis with open-source software. Moreover, we provide a method for spheroid viability quantification based on flow cytometry. The use of quantitative data from microscopy and flow cytometry in conjunction with spheroid imaging, enables the probing of normoxic, hypoxic and necrotic regions inside 3D tumor spheroids during the formation and growth of spheroids and in response to treatment. To demonstrate its feasibility, we primarily focused on the two colorectal carcinoma cell lines MC38 and SW620. Additionally, we investigated several other cancer cell lines (H1299, LCC1, MOPC, MDA-MB-231, MCF7) that show varying behavior in spheroid formation and growth. In order to visualize the hypoxic core in real-time with fluorescent microscopy, cell lines were transduced with a functionalized HIF-reporter construct (HBR-6U), which is widely applied for the determination of tumor hypoxia [13], [14], [15], [16]. Other functionalized reporter constructs could also be integrated into the presented approach. The fluorescent dyes Hoechst and propidium iodide (PI) were used as general DNA stain and viability marker, respectively.

## Materials and methods

### 2D cell culture

The human and mouse colorectal carcinoma cell lines, SW620 (a gift from Janine T. Erler, University of Copenhagen) and MC38 (a gift from Lubor Borsig, University of Zurich), respectively, were cultured in DMEM (Gibco; 41966–029) supplemented with 10 % (v/v) fetal bovine serum (Gibco; 10270–106); 1 % (v/v) penicillin–streptomycin (Gibco; 15140122). Every 3–4 months, cell lines were tested for mycoplasma contamination (MycoAlert, Lonza). Cells were cultured in a 5 % CO_2_ humified atmosphere at 37 °C. Additional cell lines (H1299, LCC1, MOPC, MDA-MB-231, MCF7) were tested for the formation of spheroids with hypoxic core as well; all details are provided in the Supplementary Document.

### Lentiviral transduction

To produce hypoxia sensing cell lines, cells were transduced with a hypoxia inducible factor (HIF) reporter construct which drives the expression of enhanced yellow fluorescent protein (eYFP) regulated by six HIF binding repeats (HBR-6U, Addgene plasmid #42621) [13]. Lentiviral production with the HBR-6U plasmid and transduction and cell sorting procedures are described in detail in [15].

### 3D cell culture and irradiation treatment

3D spheroids were formed by cell seeding (1000 cells for MC38 and 5000 cells for SW620 per well (in 200uL) in Nucleon^TM^ Sphera^TM^ 96-well plates (Thermo Fischer Scientific). The plates were centrifuged for 5 min at 500 x *g* and afterwards incubated in a 5 % CO_2_ humified atmosphere at 37 °C. Information about spheroid formation for the additional cell lines tested is given in the Supplementary Material. Three days after seeding, spheroids were irradiated with an RS-2000 225 kV irradiator (Rad Source) at 6.63 Gy/minute.

### Fluorescent microscopy

Spheroids were imaged using a Leica Thunder Imager 3D Live Cell widefield system on the DMi8 platform (Leica Microsystems AG) equipped with a Leica monochrome fluorescence DFC9000 GTC sCMOS camera and a Leica LED8 fluorescence light source. A 5 × Leica N Plan PH0 air objective (NA = 0.12) was used, with a pixel size of 1.29 μm in X and Y. Images were acquired in 2D mode, manually focused, and saved in 16-bit format using Leica LAS X software. 6 h before image acquisition, Hoechst 33,342 (MedChemExpress, HY-15559A, 2 µg/mL) and propidium iodide (MedChemExpress, HY-D0815, 5 µg/mL) were added to the spheroids. To avoid toxicity from prolonged PI exposure, separate samples were imaged at consecutive time points. For Hoechst, excitation was set to 390 nm at 80 % LED power with a 460/80 nm emission filter and 400 ms exposure. Propidium iodide imaging used 555 nm excitation at 60 % LED power with a 642/80 nm emission filter and 400 ms exposure. For eYFP, 510 nm excitation at 60 % LED power and a 535/70 nm emission filter were used with 400 ms exposure. Imaging targeted the central plane of the spheroid, approximately 300 to 400 μm deep, using a single focal plane to ensure consistent visualization. Spheroid diameters ranged from 600 to 800 μm on the day of analysis.

### Imaging analysis with custom-written script

Spheroid images were analyzed using the open-source software Fiji-ImageJ with a new specially composed custom-written script that is provided in the supplementary data. Huang thresholding [17] was used to mask the region of interest (ROI) of the total spheroid (Hoechst), the hypoxic core (eYFP) and the necrotic core (PI). Within the ROI, the area and integrated density of each fluorophore is measured as well as the mean gray value of the background in each channel. The total volume was calculated from the area-equivalent diameter of the area of interest (Hoechst, eYFP or PI). Data was presented either by displaying the volumes of each ROI or by showing the background corrected integrated intensity of PI or eYFP per spheroid volume.

### Flow cytometry of single spheroids

To dissociate the spheroids into single cell suspensions, medium was carefully aspirated from the well and 110 µL of 0.25 % trypsin (Gibco; 15090046) was added. The spheroids were incubated for up to 40 min at 37⁰C. Depending on the spheroid density, additional pipetting was occasionally needed every 10–15 min to dissociate the spheroid. Upon spheroid disintegration, trypsin was neutralized by adding 110 µL of medium. Cell suspension of each single spheroid was filtered into flow cytometry tubes and centrifuged at 600 x *g* for 5 min. After supernatant aspiration, cells were stained with Zombie NIR^TM^ dye (1:500 in PBS, BioLegend) and analyzed by flow cytometry (FACS Canto II analyzer, BD Biosciences). Data was analyzed using FlowJo software v10.7.2.

### Statistical analysis

GraphPad Prism v9.5.1. was used to analyze the data applying two-tailed unpaired *t*-test and one-way ANOVA with Dunnett’s correction for multiple comparisons. For all experiments: *, P < 0.05; **, P < 0.01; ***, P < 0.001; ****, P < 0.0001.

## Results and discussion

**Fig. 1:**
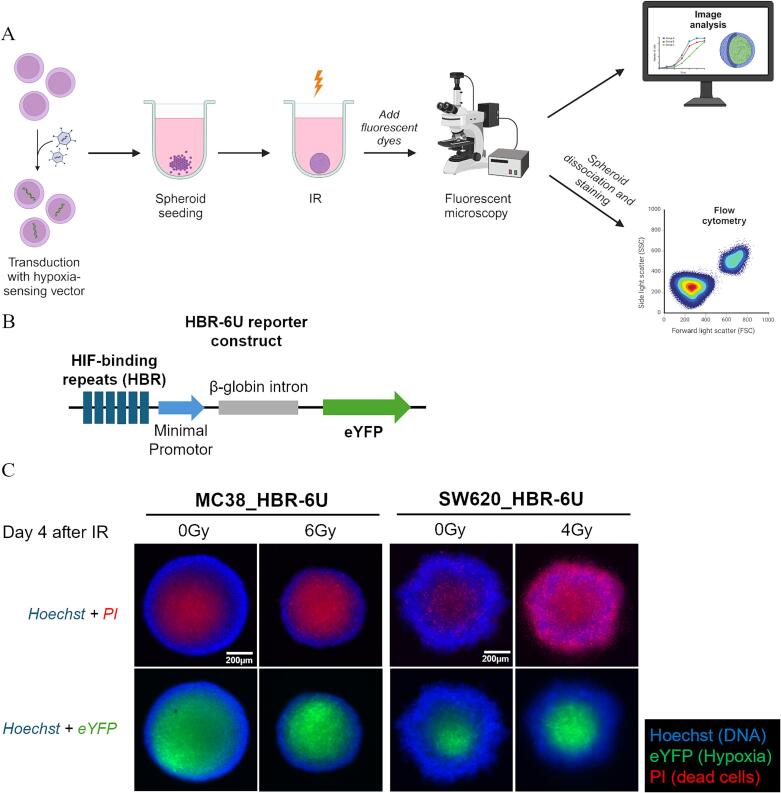
Protocol overview for spheroid seeding, fluorescent live-cell microscopy, and analysis. (A) Schematic overview of the workflow (created with Biorender.com). (B) Map of the HIF-reporter construct HBR-6U [13]. (C) Live-cell imaging of unirradiated and irradiated MC38_HBR-6U and SW620_HBR-6U spheroids show a hypoxic (eYFP-expressing) core with an inner necrotic (PI-stained) region 4 days after treatment. IR, ionizing radiation; PI, propidium iodide; eYFP, enhanced yellow fluorescent protein. (For interpretation of the references to color in this figure legend, the reader is referred to the web version of this article.)

Here, we describe a straightforward and widely applicable workflow for seeding, imaging, and quantification of spheroids in radiotherapy and hypoxia-related research (Fig. 1A). Ultra-low attachment (ULA) plates provide a highly reproducible and easy to monitor tool for spheroid generation [1]. Imaging spheroids with fluorescent live-cell microscopy has the advantage of being less cumbersome and time consuming than e.g., cryosectioning followed by immunocytochemistry. In addition, spheroids can be used for quantitative analysis by flow cytometry subsequent to the analysis with imaging techniques. To visualize formation of a hypoxic core during spheroid growth in situ, cells were transduced with a HIF-reporter construct (Fig. 1B) [13]. Of note, HIF-signaling can in part be induced also in response to other stress factors [6], [18], [19], nevertheless this reporter construct is widely accepted to probe the dynamics of hypoxia [13], [14], [15]. For validation, we compared eYFP expression in MC38_HBR6U spheroids against immunohistochemical staining of the gold-standard exogenous hypoxia marker pimonidazole in spheroids derived from MC38 non-transduced cells (Supplementary Fig. 4). Upon PI staining, tumor cell viability can be visualized throughout the spheroid. Interestingly irradiation may have markedly distinct effects on spheroids derived from different cell lines, which can be directly visualized by this approach. For example, MC38-derived spheroids showed an increase in the necrotic core and a reduction in spheroid size upon IR, while IR of SW620-derived spheroids predominantly killed cells in the outer proliferating rim of the spheroid (Fig. 1C, Supplementary Fig. 1).

An important aspect of using spheroids in preclinical studies is the ability to quantify the composition of the spheroids and their growth behavior. With the use of microscopy images, spatially separated regions within a spheroid can be identified, enabling the investigator to quantify different treatment responses. Here, we composed a new script written in the ImageJ Macro language (IJM), that was used to access and control all functions of the open-source Fiji-ImageJ image processing software necessary to quantitatively analyze different regions-of-interest within spheroids. The script is provided as .IJM file, as well as text file with additional color-coded information in the Supplementary Material. Even though quantitative 3D data must be extrapolated from 2D images, thereby only providing relative rather than absolute numbers on viability and hypoxia, this script constitutes a straightforward tool for the determination of selected endpoints of interest in respective 3D spheroid models. An alternative approach to circumvent the need for 2D extrapolation would be to acquire stacked images using confocal microscopy and subsequently reconstruct the 3D spheroid. However, full visualization of the cells at the core of the spheroid was compromised due to spheroid size and density, which exceeds the optimal z-stack imaging range of confocal microscopy (data not shown).

**Fig. 2:**
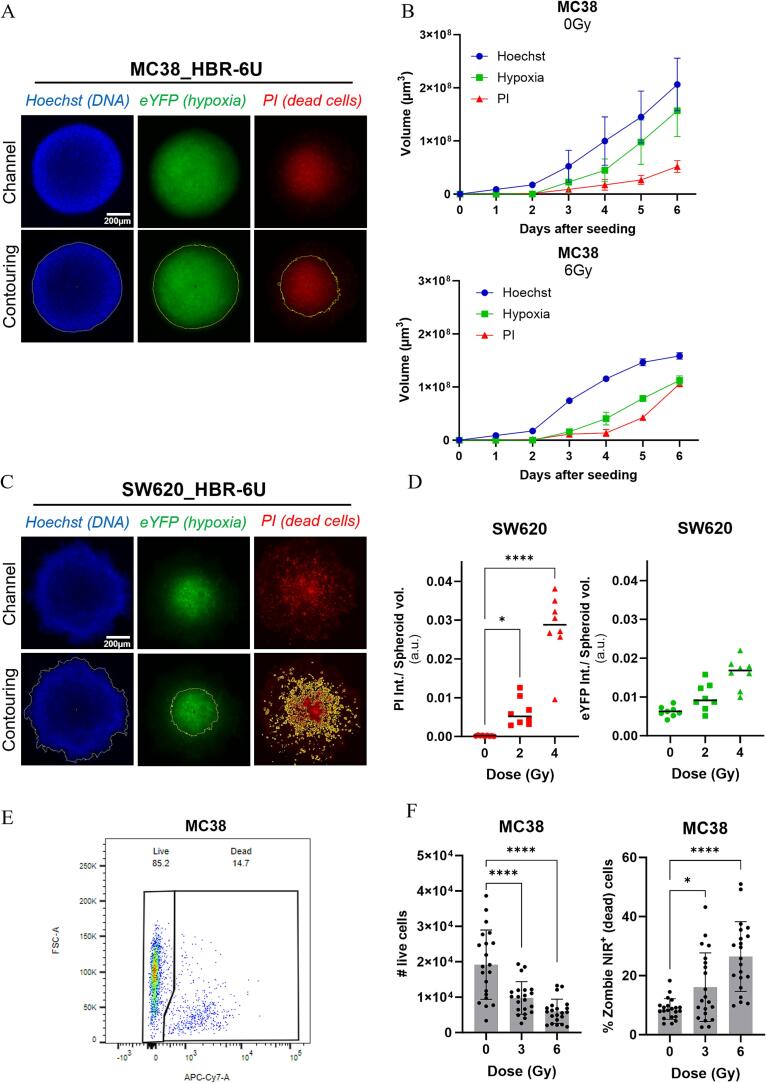
Spheroid analysis methods based on image processing or flow cytometry. (A, C) Contouring method of the total spheroid (Hoechst), the hypoxic (eYFP) and necrotic (PI) core based on Huang thresholding in MC38_HBR-6U (A) and SW620_HBR-6U spheroids (C). (B) MC38_HBR-6U spheroid volume growth curve after 0 and 6 Gy of IR over 6 days. (D) Quantification of the fluorescent intensity of eYFP or PI over the total spheroid volume (PI int./spheroid vol.) three days after treatment with different doses (0, 2, 4 Gy) of IR (left panel, see also [15]). (E) Gating strategy for live/dead staining in single spheroid flow cytometry analysis. (F) Effect on MC38 spheroid viability 3 days after treatment with different doses of IR (0, 3, 6 Gy) assessed with flow cytometry (one data point per spheroid). Results represent the mean ± SD of three independent experiments.

The total spheroid, as well as the hypoxic and necrotic core, were almost perfect spheres in MC38-derived spheroids, facilitating contouring of respective areas (Fig. 2A, Supplementary Fig. 2A), and quantification of the volume from the area-equivalent diameter. Thereby, spheroid growth and the impact of IR can be carefully monitored over several days (Fig. 2B, Supplementary Fig. 2A). For SW620 spheroids, the necrotic area in the core was not homogeneous, so the script was adapted to sum up the intensities of all individual necrotic and/or hypoxic cells and to plot this value as ratio of the entire spheroid volume (as assessed by Hoechst contouring) (Fig. 2C and D, Supplementary Fig. 2B). Furthermore, it is also possible to apply the contouring mask related to an area of interest x (i.e. endpoint x) for the quantification of the endpoint y within this defined area of interest x. For example, this approach would allow to quantify cell death (endpoint y) specifically within the hypoxic core of the spheroids (area of interest x).

Using flow cytometry analysis of dissociated spheroids, the absolute amount and percentage of live and dead cells can be determined and thereby complement image-based data analysis of the same spheroids. In our hands, single cell suspension staining with Zombie NIR viability dye was superior to PI-based staining, most probably due to photobleaching of the PI dye during image acquisition and sample preparation for flow cytometry (Fig. 2E, F). Of note, care must be taken prior to spheroid collection for flow cytometry analysis as dead cells in the periphery might dissociate from the spheroid. An approximate 3-fold increase was observed after 6 Gy of IR, both in the volume of the PI-stained spheroid core based on microscopy imaging and in the percentage in Zombie NIR^+^ cells recorded by flow cytometry. This indicates a robust correlation between image quantification using our custom-written script and flow cytometry data (Supplementary Fig. 3).

In summary, we developed a straightforward and widely applicable workflow and protocol for spheroid seeding, fluorescent live-cell microscopy and quantification based on image-analysis with a ready-to-use script and flow cytometry. Using functionalized reporters, spatially distinct regions can be identified during spheroid growth and in response to treatment, which is of great relevance in hypoxia- and radiotherapy-related research. Importantly, due to the variance in size, shape, density and texture of spheroids derived from different cell lines, each step must be carefully evaluated and might need adjustments. In this context, the image-analysis script is adaptable and can be extended to accommodate additional quantification parameters as needed.

## CRediT authorship contribution statement

**Claire Beckers:** Conceptualization, Formal analysis, Investigation, Methodology, Resources, Visualization, Writing – original draft, Writing – review & editing. **Lazaros Vasilikos:** Conceptualization, Formal analysis, Investigation, Methodology. **Lorena Moor:** Investigation, Methodology. **Martin Pruschy:** Conceptualization, Funding acquisition, Resources, Supervision, Writing – original draft, Writing – review & editing.

## Funding

This project has received funding from the European Union’s Horizon 2020 research and innovation program under the Marie Sklodowska-Curie grant agreement No 860245 and the Swiss National Science Foundation (310030_215674).

## Declaration of competing interest

The authors declare that they have no known competing financial interests or personal relationships that could have appeared to influence the work reported in this paper.
