# Supplementary material for: Live-cell imaging and analysis of 3D spheroids in hypoxia- and radiotherapy-related research
Source: Clin Transl Radiat Oncol. 2025 Jan 15;51:100920. doi: 10.1016/j.ctro.2025.100920 (PMC11787710; doi:10.1016/j.ctro.2025.100920)
Supplement: Supplementary Data 1 [file mmc1.docx]

**Supplementary**

**Material & Methods**

**3D cell culture of additional cell lines**

H1299 (a gift from Johannes Häberle, University of Zurich) were cultured in RPMI (Gibco; 11875093), LCC1 and MOPC (a gift from Verena Jendrossek, University of Duisburg-Essen) in DMEM (Gibco; 41966-029) and F12 (Gibco; 11765-054) respectively, and MCF7 and MDA-MB-231 (a gift from Pierre Sonveaux, UCLouvain) in DMEM (Gibco; 41966-029 and 41965-039 respectively) supplemented with 10% (v/v) fetal bovine serum (Gibco; 10270-106); 1% (v/v) penicillin-streptomycin (Gibco; 15140122). 3D spheroids were formed by seeding 10 000 cells/well for H1299, 4000 cells/well for LCC1, 5000 cells/well for MOPC, MCF7 and MDA-MB-231 (200uL) in Nucleon^TM^ Sphera^TM^ 96-well plates (Thermo Fischer Scientific). The plates were centrifuged for 5min at 500 x *g* and afterwards incubated in a 5% CO_2_ humified atmosphere at 37°C. For MCF7 and MDA-MB-231, 15µg/mL of collagen I (Gibco; A1048301) was added 24h after seeding to support spheroid formation. Images were acquired six days after seeding.

**Cryosectioning spheroids**

MC38 spheroids were treated with pimonidazole (500μM; Hypoxyprobe, US) for 3.5h. Thereafter, spheroids were fixed in 4% PFA for 2 hours, washed with PBS and incubated in a 30% sucrose (in PBS) solution overnight. Spheroids were mounted in OCT (Thermo Fisher Scientific) embedding medium and frozen on dry ice. Cryosections of 20μm were made with a cryostat CryoStar NX70 (Thermo Fisher Scientific), mounted onto slides and stored at -80°C. Sections were rehydrated in 0.1% PBS-Tween for 10 minutes, permeablized with 0.3% Triton X in PBS for 8min and blocked with 3% bovine serum albumin (BSA) in PBS for 2 hours at room temperature. Sections were incubated with APC-conjugated anti-pimonidazole rat antibody (Hypoxyprobe, US) at a dilution of 1:100 in 3% BSA in PBS overnight at 4°C. Before mounting, sections were washed twice with PBS for 10min and were stained with 5μg/mL Hoechst 33342 (MedChemExpress) for 15min at room temperature. For MC38-HBR-6U spheroids, the procedure was the same, excluding the pimonidazole treatment and incubation with anti-pimonidazole antibody. Images were taken with a Leica Thunder Imager widefield microscope at 10x magnification. For APC, excitation was set to 635nm with a 642/80nm emission filter, for eYFP, excitation was set to 510nm with a 535/70nm emission filter.

**Adaptable ImageJ script for quantitative image-analysis**

The ImageJ script provides data needed for quantitative analysis in a results table: area, mean gray value, mean intensity and integrated density for the spheroid and background in every channel. The following formulas are applied to calculate the area-equivalent diameter (D_e_), volume (V) and total corrected cell fluorescence (TCCF) for every channel (Hoechst, PI, eYFP). TCCF is used to correct for the background intensity, that may differ from channel to channel and also among individual images [1].

Area-equivalent diameter (D_e_) = $\sqrt{4*area/\pi}$

Volume (V) =$\frac{\pi*D_{e}^{3}}{6}$

Total corrected cell fluorescence (TCCF) =
𝐼𝑛𝑡𝑒𝑔𝑟𝑎𝑡𝑒𝑑 𝑑𝑒𝑛𝑠𝑖𝑡𝑦 𝑜𝑓 𝑠𝑝ℎ𝑒𝑟𝑜𝑖𝑑 ∗ (𝑆𝑝ℎ𝑒𝑟𝑜𝑖𝑑 𝑎𝑟𝑒𝑎 ∗ 𝑀𝑒𝑎𝑛 𝑖𝑛𝑡𝑒𝑛𝑠𝑖𝑡𝑦 𝑏𝑎𝑐𝑘𝑔𝑟𝑜𝑢𝑛𝑑)

| **Legend^*^** | **Feature** | **Adaptability** |
| --- | --- | --- |
| File names | Name of raw image file | Adapt to name used |
| Saving path | Place where image should be stored | Adapt to desired location |
| Brightness level | Brightness and contrast of a channel | Adapt empirically to increase brightness and contrast but avoid saturation. Make sure the same settings are applied within one data set. |
| Huang Threshold | Differentiates which pixels of the image belong to the object (spheroid) and which to the background | Define empirically at the start of image analysis; typically, a value around 20-30% of the highest intensity measured as the minimal intensity is a good starting point. However, this changes from cell line to cell line. The intensity profile in Image J (Analyze 🡪 plot profile) is a useful tool for estimation of the threshold. Make sure the same settings are applied within one data set. |
| Minimal size included in mask | Defines the size of particles that should be included in the mask, lower sized particles are excluded. | Define empirically based on the form and size of the spheroid. Only useful for uniform areas (e.g., PI staining in MC38), in non-uniform areas (e.g., PI staining in SW620) all particles should be included. Make sure the same settings are applied within one data set. |

*Color code corresponds to adaptable features in ImageJ script.

1. **ImageJ script for MC38_HBR-6U spheroids**

1. run("Stack to Images");

2. selectWindow("MC38_0Gy_A1-0001");

3. rename("BF")

4. saveAs("Tiff", "C:/Users/Desktop/Spheroids/BF.tif");

5. close();

6.

7. selectWindow("MC38_0Gy_A1-0002");

8. rename("Hoechst")

9. call("ij.ImagePlus.setDefault16bitRange", 16);

10. setMinAndMax(0, 43000);

11. run("Apply LUT");

12. selectWindow("MC38_0Gy_A1-0003");

13. rename("PI")

14. call("ij.ImagePlus.setDefault16bitRange", 16);

15. setMinAndMax(0, 35000);

16. run("Apply LUT");

17. selectWindow("MC38_0Gy_A1-0004");

18. rename("Hypoxia")

19. call("ij.ImagePlus.setDefault16bitRange", 16);

20. setMinAndMax(0, 40000);

21. run("Apply LUT");

22.

23. selectWindow("Hoechst");

24. run("Duplicate...", " ");

25. setAutoThreshold("Huang");

26. //run("Threshold...");

27. setThreshold(12000, 65535, "raw");

28. setOption("BlackBackground", false);

29. run("Convert to Mask");

30. run("Analyze Particles...", "size=18000-Infinity show=Masks add");

31. selectWindow("Mask of Hoechst-1");

32. selectWindow("Hoechst");

33. roiManager("Select", 0);

34. run("RGB Color");

35. run("Measure");

36.

37. run("From ROI Manager");

38. run("Duplicate...", " ");

39. saveAs("Jpeg", " C:/Users/Desktop/Spheroids/Hoechst-Mask.jpg");

40. close();

41.

42. //setTool("rectangle");

43. makeRectangle(1404, 192, 102, 87);

44. run("Measure");

45. roiManager("Deselect");

46. roiManager("Delete");

47. selectWindow("Mask of Hoechst-1");

48. close();

49. selectWindow("Hoechst-1");

50. close();

51.

52. selectWindow("Hypoxia");

53. run("Duplicate...", " ");

54. setAutoThreshold("Huang");

55. //run("Threshold...");

56. setThreshold(6000, 65535, "raw");

57. setOption("BlackBackground", false);

58. run("Convert to Mask");

59. run("Analyze Particles...", "size=15000-Infinity show=Masks add");

60. selectWindow("Mask of Hypoxia-1");

61. selectWindow("Hypoxia");

62. roiManager("Select", 0);

63. run("RGB Color");

64. run("Measure");

65.

66. run("From ROI Manager");

67. run("Duplicate...", " ");

68. saveAs("Jpeg", "C:/Users/Desktop/Spheroids/Hypoxia-Mask.jpg");

69. close();

70.

71. //setTool("rectangle");

72. makeRectangle(1404, 192, 102, 87);

73. run("Measure");

74. roiManager("Deselect");

75. roiManager("Delete");

76. selectWindow("Mask of Hypoxia-1");

77. close();

78. selectWindow("Hypoxia-1");

79. close();

80.

81. selectWindow("PI");

82. run("Duplicate...", " ");

83. setAutoThreshold("Huang");

84. //run("Threshold...");

85. setThreshold(5500, 65535, "raw");

86. setOption("BlackBackground", false);

87. run("Convert to Mask");

88. run("Analyze Particles...", "size=10000-Infinity show=Masks add");

89. selectWindow("Mask of PI-1");

90. selectWindow("PI");

91. roiManager("Select", 0);

92. run("RGB Color");

93. run("Measure");

94.

95. run("From ROI Manager");

96. run("Duplicate...", " ");

97. saveAs("Jpeg", "C:/Users/Desktop/Spheroids/PI-Mask.jpg");

98. close();

99.

100. //setTool("rectangle");

101. makeRectangle(1404, 192, 102, 87);

102. run("Measure");

103. roiManager("Deselect");

104. roiManager("Delete");

105. selectWindow("Mask of PI-1");

106. close();

107. selectWindow("PI-1");

108. close();

109.

110. selectWindow("PI");

111. run("Merge Channels...", "c1=PI c3=Hoechst create keep");

1120. run("RGB Color");

113. run("Scale Bar...", "width=200 height=12 font=42 color=White

_background=None location=[Lower Right] bold");

114. saveAs("Tiff", "C:/Users/Desktop/Spheroids/Hoechst, PI.tif");

115. run("Merge Channels...", "c1=PI c3=Hoechst c2=Hypoxia create keep");

116. run("RGB Color");

117. run("Scale Bar...", "width=200 height=12 font=42 color=White

_background=None location=[Lower Right] bold");

118. saveAs("Tiff", "C:/Users/Desktop/Spheroids/Hoechst, PI, Hypoxia.tif");

119. run("Merge Channels...", "c3=Hoechst c2=Hypoxia create keep");

120. run("RGB Color");

121. run("Scale Bar...", "width=200 height=12 font=42 color=White

_background=None location=[Lower Right] bold");

122. saveAs("Tiff", "C:/Users/Desktop/Spheroids/Hoechst, Hypoxia.tif");

123.

124. selectWindow("Hoechst");

125. saveAs("Tiff", "C:/Users/Desktop/Spheroids/Hoechst.tif");

126. selectWindow("PI");

127. saveAs("Tiff", "C:/Users/Desktop/Spheroids/PI.tif");

128. selectWindow("Hypoxia");

129. saveAs("Tiff", "C:/Users/Desktop/Spheroids/Hypoxia.tif");

130. selectWindow("Hoechst, PI.tif");

131. close();

132. selectWindow("Hoechst, PI, Hypoxia.tif");

133. close();

134. selectWindow("Hoechst, Hypoxia.tif");

135. close();

136. selectWindow("PI.tif");

137. close();

138. selectWindow("Hypoxia.tif");

139. close();

140. selectWindow("Hoechst.tif");

1. **ImageJ script for SW620_HBR-6U spheroids**

1. run("Stack to Images");

2. selectWindow("SW620_0Gy_A1-0001");

3. rename("BF")

4. saveAs("Tiff", "C:/Users/Desktop/Spheroids/BF.tif");

5. close();

6.

7. selectWindow("SW620_0Gy_A1-0002");

8. rename("Hoechst")

9. call("ij.ImagePlus.setDefault16bitRange", 16);

10. setMinAndMax(0, 43000);

11. run("Apply LUT");

12. selectWindow("SW620_0Gy_A1-0003");

13. rename("PI")

14. call("ij.ImagePlus.setDefault16bitRange", 16);

15. setMinAndMax(0, 10000);

16. run("Apply LUT");

17. selectWindow("SW620_0Gy_A1-0004");

18. rename("Hypoxia")

19. call("ij.ImagePlus.setDefault16bitRange", 16);

20. setMinAndMax(0, 60000);

21. run("Apply LUT");

22.

23. selectWindow("Hoechst");

24. run("Duplicate...", " ");

25. setAutoThreshold("Huang");

26. //run("Threshold...");

27. setThreshold(12000, 65535, "raw");

28. setOption("BlackBackground", false);

29. run("Convert to Mask");

30. run("Analyze Particles...", "size=18000-Infinity show=Masks add");

31. selectWindow("Mask of Hoechst-1");

32. selectWindow("Hoechst");

33. roiManager("Select", 0);

34. run("RGB Color");

35. run("Measure");

36.

37. run("From ROI Manager");

38. run("Duplicate...", " ");

39. saveAs("Jpeg", "C:/Users/Desktop/Spheroids/Hoechst-Mask.jpg");

40. close();

41.

42. //setTool("rectangle");
43. makeRectangle(1404, 192, 102, 87);
44. run("Measure");
45. roiManager("Deselect");
46. roiManager("Delete");
47. selectWindow("Mask of Hoechst-1");
48. close();
49. selectWindow("Hoechst-1");
50. close();
51.
52. selectWindow("Hypoxia");
53. run("Duplicate...", " ");
54. setAutoThreshold("Huang");
55. //run("Threshold...");
56. setThreshold(5000, 65535, "raw");
57. setOption("BlackBackground", false);
58. run("Convert to Mask");
59. run("Analyze Particles...", "size=15000-Infinity show=Masks add");
60. selectWindow("Mask of Hypoxia-1");
61. selectWindow("Hypoxia");
62. roiManager("Select", 0);
63. run("RGB Color");
64. run("Measure");
65.
66. run("From ROI Manager");
67. run("Duplicate...", " ");
68. saveAs("Jpeg", "C:/Users/Desktop/Spheroids/Hypoxia-Mask.jpg");
69. close();
70.
71. //setTool("rectangle");
72. makeRectangle(1404, 192, 102, 87);
73. run("Measure");
74. roiManager("Deselect");
75. roiManager("Delete");
76. selectWindow("Mask of Hypoxia-1");
77. close();
78. selectWindow("Hypoxia-1");
79. close();
80.
81. selectWindow("PI");
82. run("Duplicate...", " ");
83. setAutoThreshold("Huang");
84. //run("Threshold...");
85. setThreshold(5200, 65535, "raw");
86. setOption("BlackBackground", false);
87. run("Convert to Mask");
88. run("Analyze Particles...", "show=Masks add");
89.
90. selectWindow("Mask of PI-1");
91. selectWindow("PI");
92.
93. count = roiManager("count");
94. array = newArray(count);
95. for (i=0; i<array.length; i++) {
96. array[i] = i;}
97.
98. roiManager("select", array);
99. roiManager("Combine");
100. run("RGB Color");
101. run("Measure");
102.
103. run("From ROI Manager");
104. run("Duplicate...", " ");
105. saveAs("Jpeg", "C:/Users/Desktop/Spheroids/PI-Mask.jpg");
106. close();
107.
108. //setTool("rectangle");
109. makeRectangle(1404, 192, 102, 87);
110. run("Measure");
111. roiManager("Deselect");
112. roiManager("Delete");
113. selectWindow("Mask of PI-1");
114. close();
115. selectWindow("PI-1");
116. close();
117.
118. selectWindow("PI");
119. run("Merge Channels...", "c1=PI c3=Hoechst create keep");
120. run("RGB Color");
121. run("Scale Bar...", "width=200 height=12 font=42 color=White
background=None _location=[Lower Right] bold");
122. saveAs("Tiff", "C:/Users/Desktop/Spheroids/Hoechst, PI.tif");
123. run("Merge Channels...", "c1=PI c3=Hoechst c2=Hypoxia create keep");
124. run("RGB Color");
125. run("Scale Bar...", "width=200 height=12 font=42 color=White
background=None _location=[Lower Right] bold");
126. saveAs("Tiff", "C:/Users/Desktop/Spheroids/Hoechst, PI, Hypoxia.tif");
127. run("Merge Channels...", "c3=Hoechst c2=Hypoxia create keep");
128. run("RGB Color");
129. run("Scale Bar...", "width=200 height=12 font=42 color=White
background=None _location=[Lower Right] bold");
130. saveAs("Tiff", "C:/Users/Desktop/Spheroids/Hoechst, Hypoxia.tif");
131.
132. selectWindow("Hoechst");
133. saveAs("Tiff", "C:/Users/Desktop/Spheroids/Hoechst.tif");
134. selectWindow("PI");
135. saveAs("Tiff", "C:/Users/ Desktop/Spheroids/PI.tif");
136. selectWindow("Hypoxia");
137. saveAs("Tiff", "C:/Users/Desktop/Spheroids/Hypoxia.tif");
138.
139. selectWindow("Hoechst, PI.tif");
140. close();
141. selectWindow("Hoechst, PI, Hypoxia.tif");
142. close();
143. selectWindow("Hoechst, Hypoxia.tif");
144. close();
145. selectWindow("PI.tif");
146. close();
147. selectWindow("Hypoxia.tif");
148. close();
149. selectWindow("Hoechst.tif");

**Suppl. Fig. 1. 3D cell culture of additional cell lines.** Live-cell imaging of H1299_HBR-6U, LCC1_HBR-6U, MOPC_HBR-6U, MCF7_HBR-6U and MDA-MB-231 spheroids showing a hypoxic (eYFP-expressing) core with an inner necrotic (PI-stained) region 6 days after seeding.

**
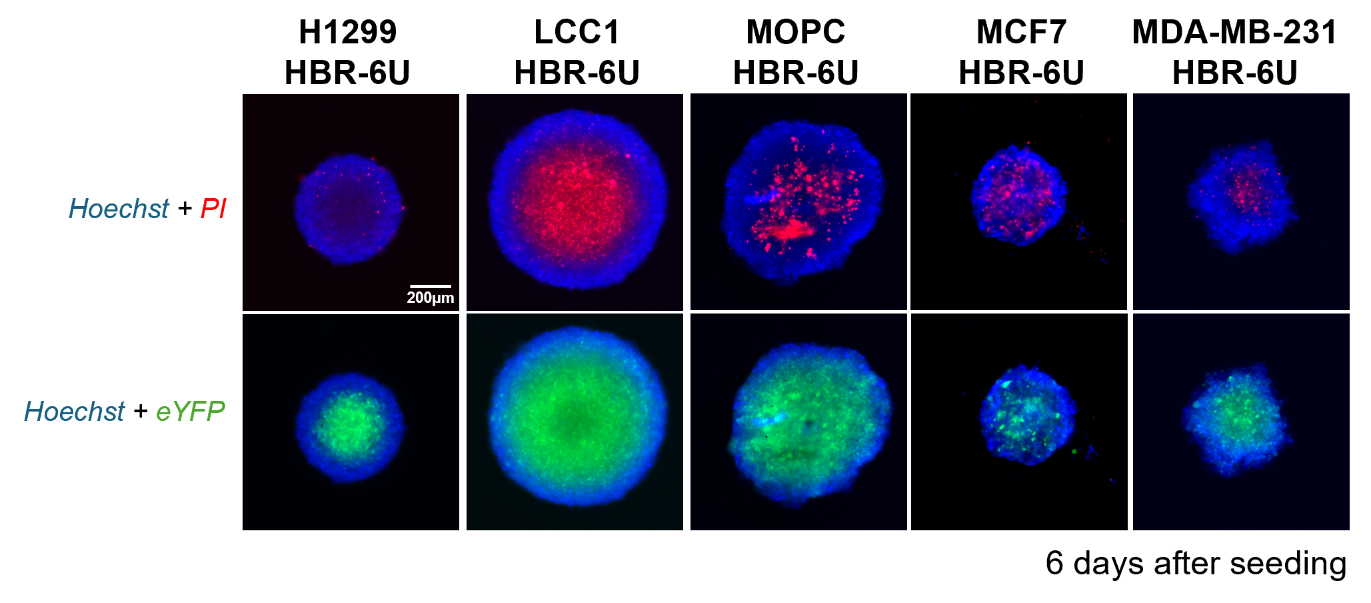
**

**Suppl. Fig. 2. Spheroid growth after IR. (A)** MC38_HBR-6U spheroid growth during six days after seeding. Spheroids were irradiated with 0 or 6Gy at day 3. **(B)** Effect of different doses of IR (0, 2 or 4Gy) on SW620_HBR-6U spheroids 3 days after treatment.


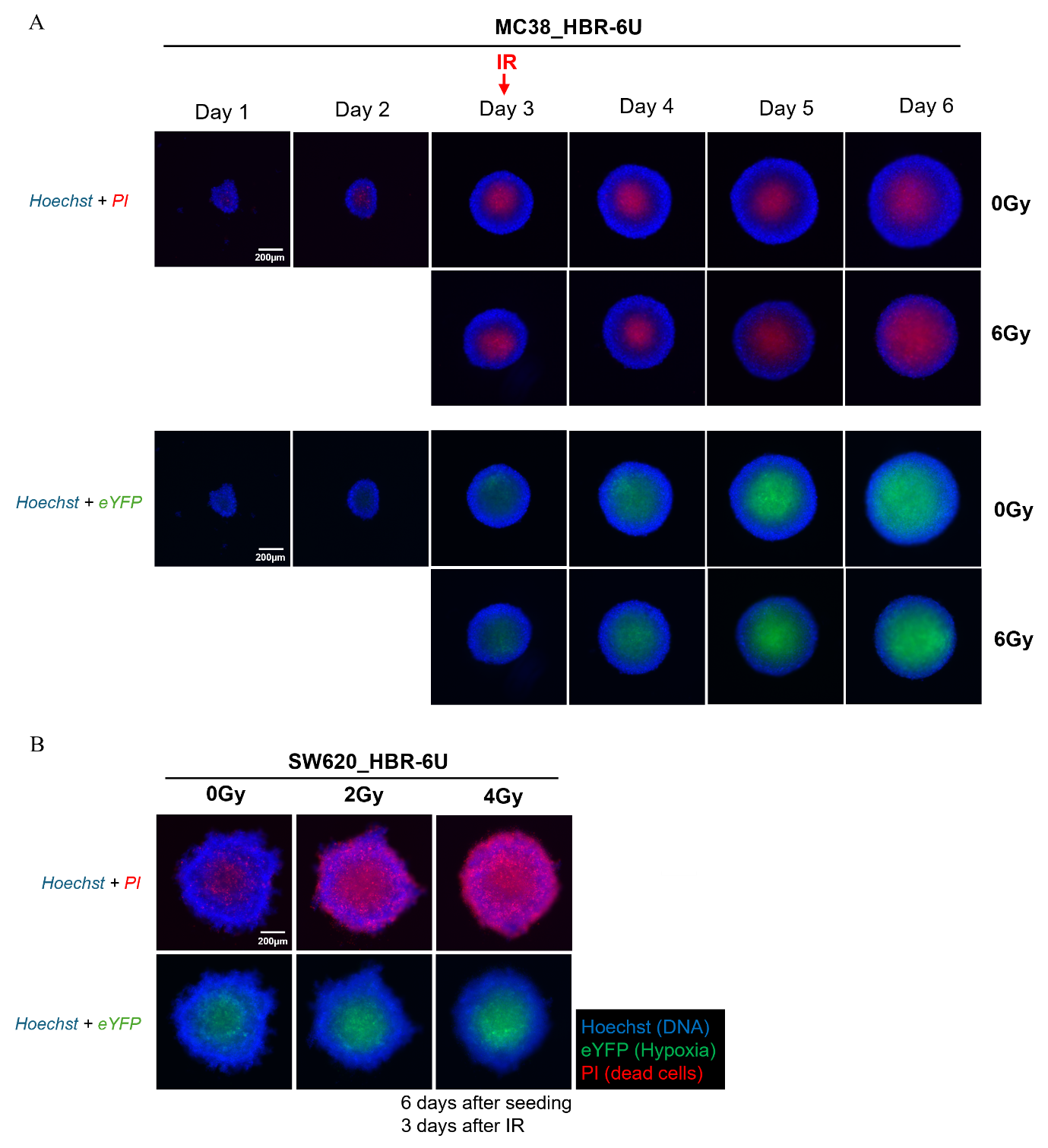

**Suppl. Fig. 3. Correlation of spheroid cell death quantification between image-based and flow cytometry analysis.** Fold-change increase in response to 6Gy of IR in **(A)** the volume of the PI-stained spheroid core based on microscopy imaging and **(B)** the percentage of Zombie NIR^+^ cells recorded by flow cytometry.

B

A

**
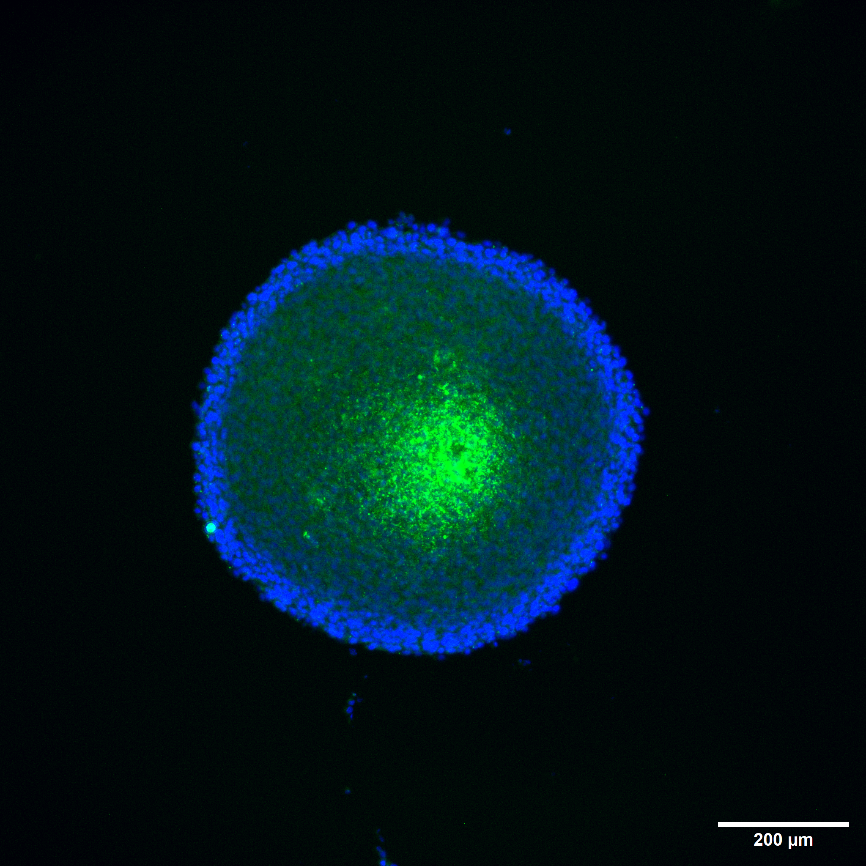
Suppl. Fig. 4. Comparative detection of pimonidazole-positive (A) and eYFP fluorescent-positive areas (B)** in MC38-spheroids and MC38-HBR-6U spheroids, respectively.

**Hoechst (DNA)**

**eYFP (Hypoxia)**


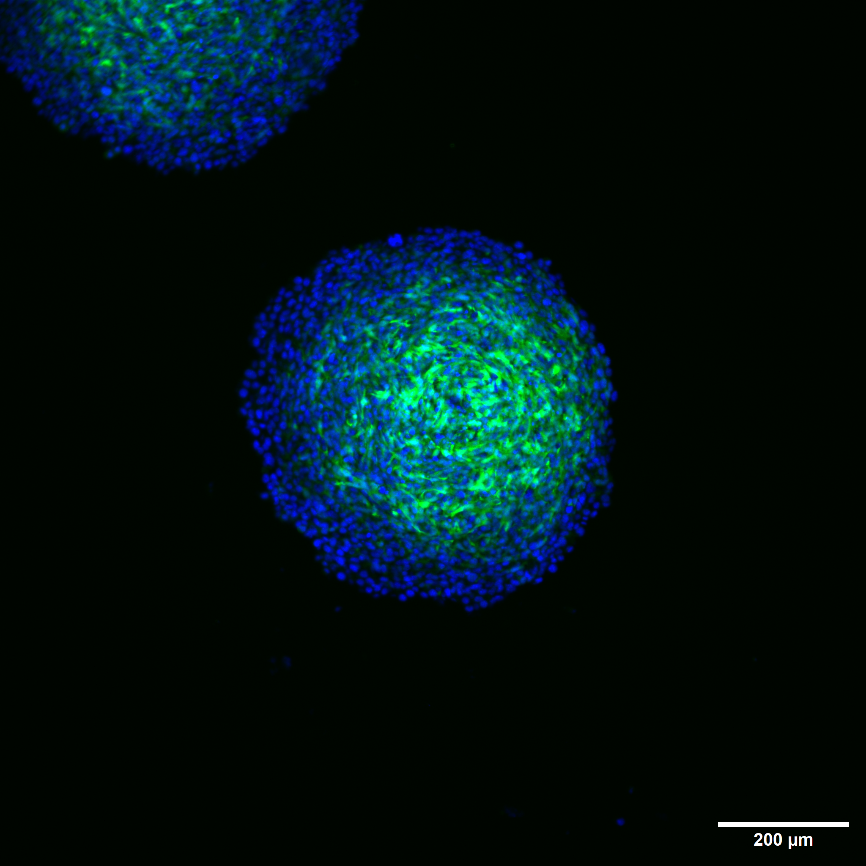

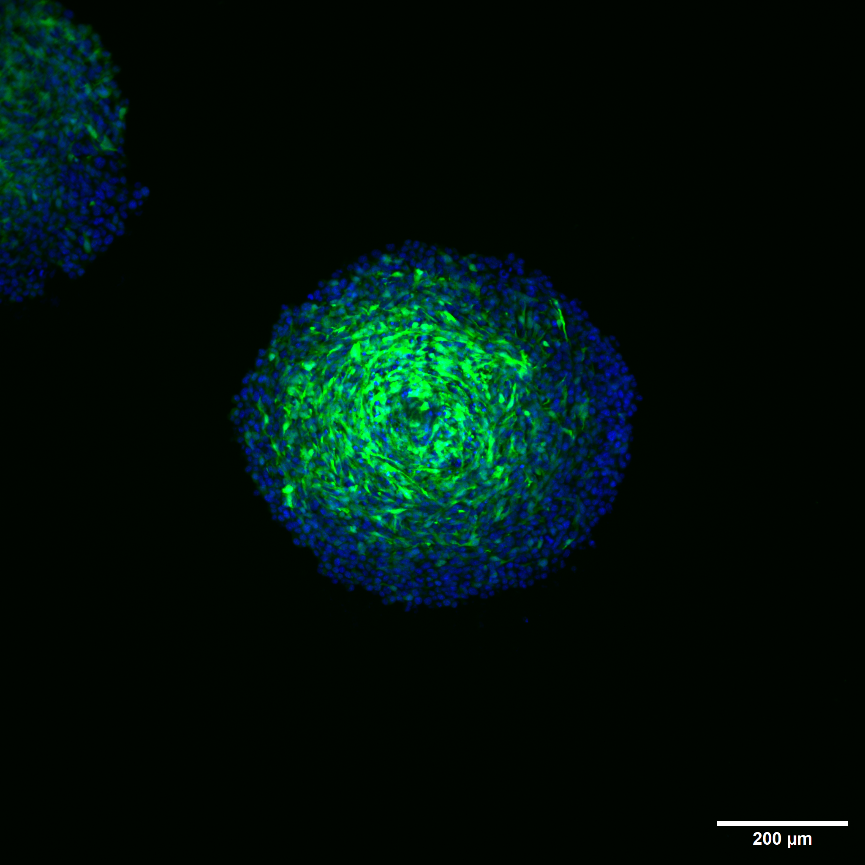


**MC38_HBR-6U**


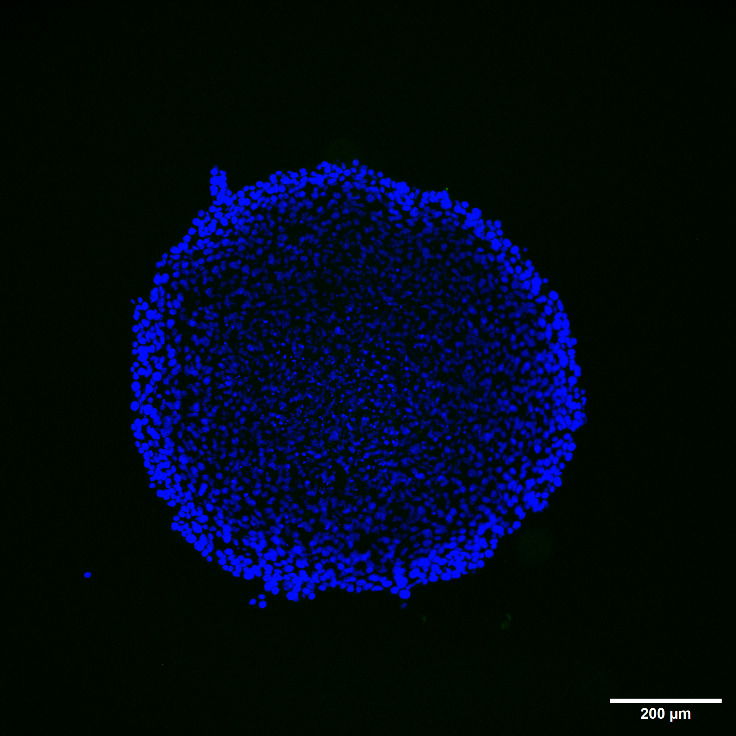

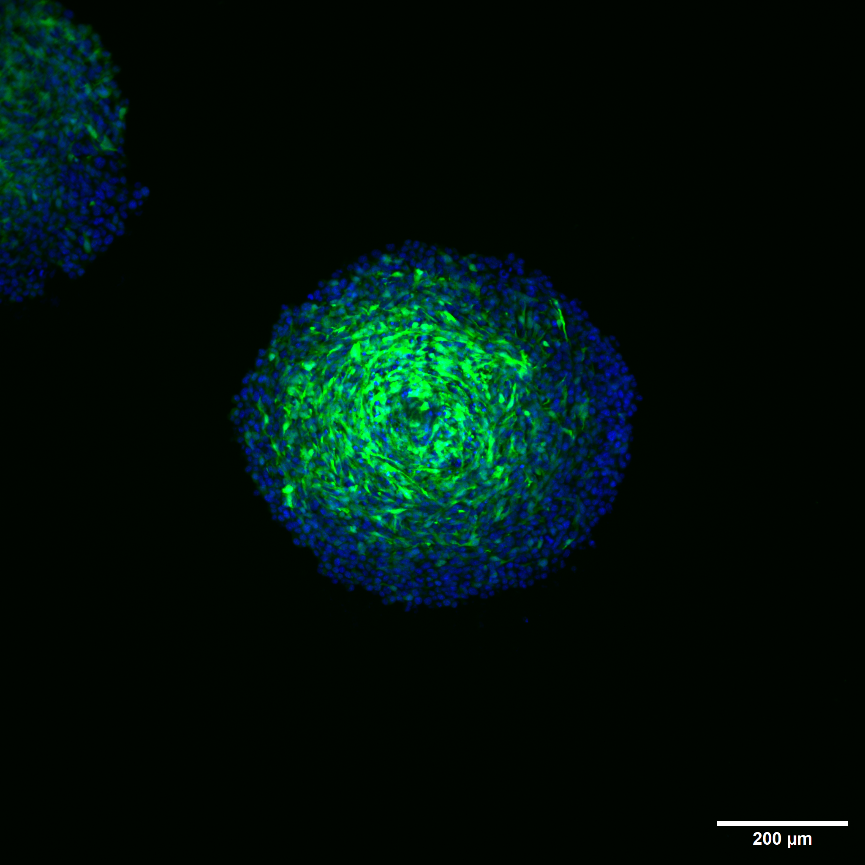


**Hoechst (DNA)**

**Pimo (Hypoxia)**

**MC38**

**CTR**

**MC38**

**Pimo**

A

B

**References**

1. Ansari, N., et al., Quantitative 3D cell-based assay performed with cellular spheroids and fluorescence microscopy. Methods Cell Biol, 2013. **113**: p. 295-309.
